# The absence of eosinophils is associated with early metastatic lesions in *Leishmania amazonensis*-infected mice

**DOI:** 10.1590/0074-02760220242

**Published:** 2024-01-08

**Authors:** Gregório Guilherme Almeida, Tassiane Assíria Martins Luehring, Pierre Henrique de Menezes Paixão, Rodrigo Pedro Soares, André Luís Branco de Barros, Rubens Lima do Monte-Neto, Wagner Luiz Tafuri, Deborah Aparecida Negrão-Corrêa, Ricardo Gonçalves

**Affiliations:** 1 Universidade Federal de Minas Gerais, Instituto de Ciências Biológicas, Departamento de Patologia Geral, Belo Horizonte, MG, Brasil; 2 Fundação Oswaldo Cruz-Fiocruz, Instituto René Rachou, Grupo de Biotecnologia Aplicada a Patógenos, Belo Horizonte, MG, Brasil; 3 Universidade Federal de Minas Gerais, Faculdade de Farmácia, Departamento de Análises Clínicas e Toxicológicas, Belo Horizonte, MG, Brasil; 4 Universidade Federal de Minas Gerais, Instituto de Ciências Biológicas, Departamento de Parasitologia, Belo Horizonte, MG, Brasil

**Keywords:** Leishmania, eosinophil, disseminated leishmaniasis

## Abstract

**BACKGROUND:**

Eosinophils are granulocytes that rapidly increase frequency in the bloodstream during helminthic infections and allergic responses. They are found in tissue infected by Leishmania during early disease, but their role during infection is not entirely understood.

**OBJECTIVES:**

We aim to compare the disease due to *Leishmania amazonensis* in BALB/c and Δdbl-GATA1 mice, which lack eosinophils.

**METHODS:**

BALB/c and Δdbl-GATA1 mice infected with *L. amazonensis* were observed for several weeks. The parasite load and dissemination pattern were assessed.

**FINDINGS:**

The Δdbl-GATA1 mice developed an anticipated dissemination of *L. amazonensis* and a worsening disease. No differences were found in the lesion development or the parasite load in the footpad among Δdbl-GATA1 mice and BALB/c eight weeks after infection. However, nine weeks after infection, massive growth of metastatic lesions appeared in several parts of the skin in Δdbl-GATA1 mice, weeks earlier than BALB/c. We observed increased parasites in the bloodstream, probably an essential dissemination route. Thirteen weeks after infection, metastatic lesions were found in all Δdbl-GATA1 mice.

**MAIN CONCLUSION:**

These results suggest a protective role of eosinophils in delaying the disease caused by *L. amazonensis*, although several limitations of this mice strain must be considered.

Leishmaniases are diseases caused by the infection with protozoan parasites belonging to the *Leishmania* genus transmitted by the bites of female phlebotomine sand flies. Infection with these parasites may cause a spectrum of clinical manifestations, which varies according to the *Leishmania* species and the host immune response.^1^ ^,^ ^2^ *Leishmania amazonensis* causes the common cutaneous leishmaniasis and, in a few cases, the anergic diffuse cutaneous leishmaniasis, which is characterised by the presence of diffuse cutaneous nodules, papules, tubercules, and infiltrated plaques and rarely causes nasopharyngeal disease. This terrible clinical form is frequently irresponsive to treatment, and patients suffer from disfiguration and social stigma.^1^

The participation of adaptive immunity guided by T cells in generating resistance against infection is well described for *Leishmania major*.^3^ ^,^ ^4^ ^,^ ^5^ However, this model does not explain susceptibility/resistance in mice infected with *L. amazonensis*, as C57BL/6 mice fail to control the infection even in the presence of IFN-γ from TH1 cells, suggesting that resistance against *L. amazonensis* would require a different immune mechanism.^6^ ^,^ ^7^

In natural conditions, transmission occurs through sand flies bites. Thus, immunity against salivary molecules may interfere with the disease’s physiopathology. Indeed, the number of eosinophils and other mononuclear phagocytes recruited to the infection site increases after repetitive sand fly bites and depends on CD4^+^ T cells signalling in response to specific salivary proteins.^8^ ^,^ ^9^ ^,^ ^10^

Eosinophils are granulocytes often increased in the bloodstream in helminth infections and allergies.^11^ In mice and humans, eosinophils compose the cellular infiltrate in tissues infected by *Leishmania* and draining lymph nodes in the early phase of the infection.^12^ ^,^ ^13^ Despite the massive presence of eosinophils in early infection, the importance of this cell during the initial steps of *Leishmania* infection has been overshadowed by a higher number of studies on macrophages, CD4^+^ T cells, and neutrophils.

To understand the role of eosinophils in several diseases, mice with a double deletion on the high-affinity GATA binding site in the GATA1 promoter (Δdbl-GATA1) were developed.^14^ They possess a defect in the maturation of eosinophils being used in several studies on allergy and eosinophil biology. However, those mice have never been studied during *Leishmania* infection. Therefore, we aimed to describe some aspects of the infection of Δdbl-GATA1 mice with *L. amazonensis*.

## MATERIALS AND METHODS

*Animals* - Six- to eight-week-old Δdbl-GATA1 mice (BALB/c background) were raised in the Biochemistry and Immunology Department - Universidade Federal de Minas Gerais (UFMG) facilities. BALB/c mice were obtained from the central animal facility of UFMG. Experiments were done in the experimental facility of the Department of Pathology. All mice were dewormed two weeks before infection and kept at day-night cycles and water and commercial chow *ad libitum*.

*Parasites* - *L. amazonensis* (IFLA/BR/1967/PH8) was cultivated in α-MEM media (Sigma) with 10% foetal calf serum (Cultilab, Brazil), 100 U/mL penicillin and 100 µg/mL streptomycin (USBiological, USA). Parasites were cultivated until the stationary phase (five - six days) in BOD shelfs at 25ºC, when they were washed by centrifugation (2000 x g, 10 min) in sterile phosphate-buffered saline (PBS). Twenty million parasites were inoculated with a 27.5 g needle intradermally into the hind footpad in a final volume of 10 µL. All *Leishmania* strains were used below five passages for infection. Lesions were measured weekly using a caliper (Pantec, USA), and footpad thickness was calculated as the difference between the uninfected and the infected footpads. A strain of *L. amazonensis* (PH8) with an integrated transfection of firefly luciferase (*L. ama*-LUC) was cultivated in the same conditions and used for bioimaging experiments.^15^

*Parasite load* - Parasite loads were accessed by a modified limiting dilution assay (LDA). Briefly, footpads or spleen were removed and homogenated with a tissue grinder with 2 mL of complete media. Homogenates were centrifuged at 100 x g for 5 min, and supernatants were transferred to another tube and centrifuged at 2200 x g for 10 min. Supernatants were discarded, and the pellet was resuspended in 1 mL of complete media and serially diluted in 96-well flat-bottom microtiter plates containing complete media. The number of viable parasites in each tissue was determined from the highest dilution at which promastigotes could be grown after 10-14 days of incubation at 25ºC.

*Evaluation of metastatic lesions* - Metastatic lesions were counted as the absolute number of new ulcers or nodular lesions in the limbs, head, and tail after eight weeks of infection. The mean number of lesions was compared between groups, as well as the percentage of mice with metastatic lesions. The development of metastatic lesions was followed by bioluminescence assay at weeks 8, 10, and 12 in mice infected with *L. ama*-LUC.^16^ Briefly, mice were anesthetised with a mixture of ketamine (80 mg/kg) and xylazine (15 mg/kg) and injected with a 75 mg/kg solution of luciferase substrate (VivoGlo, Promega) intravenously in the tail. After 20 min, bioluminescence was measured using the In Vivo Xtreme bioimager (Bruker, USA). Images were taken in high-resolution mode with 2 min exposure from a fixed-size region of interest. Results were expressed as the number of photons/s/mm^2^.

*Parasite load in the blood* - Blood (100 µL) was taken from each mouse by retro-orbital vein bleeding from week five to nine post-infection in 5 mM EDTA. DNA was extracted using the NucleoSpin Blood kit (Macherey-Nagel, USA) following the manufacturer’s protocol. Parasite load in blood was measured by quantitative polymerase chain reaction (qPCR). Briefly, 2 µL of DNA samples were mixed with 5 µL GoTaq Power Sybr Green Master Mix (Promega, USA), 400 nM of each primer (5’SSSCCMCTATWTTACACCAACCCC and 5’GGGGAGGGGCGTTCTGCGAA^17^ in a final volume of 10 µL. The PCR reaction was performed in a StepOne Plus (Applied Biosystems, USA), with the following conditions: 10 min at 95ºC initial denaturation, followed by 40 cycles of 15-sec denaturation at 95ºC and 60-sec annealing/extension at 60ºC. Melting curves were analysed to ensure reaction specificity. A 6-point 10-log standard curve was built using a known number of promastigotes (from 1 to 10^5^) diluted in 100 µL blood of uninfected mice. DNA was extracted in the same conditions described and run in the same conditions. The number of parasites/100 µL of blood/mice was estimated by interpolating the mean CT values of each animal on the curve.

*Statistical analysis* - Statistical tests used for each parameter are described within the figure’s legends. Data was organised using Microsoft Excel, and graphs, and statistical analysis was performed in Graphpad Prism (v9). Variables were tested for Gaussian distribution using the Shapiro-Wilk test. According to their distribution, Mann-Whitney U test or t-student test were used for comparisons between groups. Significance was set at p < 0.05. Bioimaging was analysed using the software Bruker Molecular Imaging (Bruker, USA).

*Ethical standards* - This work was approved by the UFMG Experimental Animal Ethics Committee (CEUA, 21/2016).

## RESULTS

**Fig. 1:**
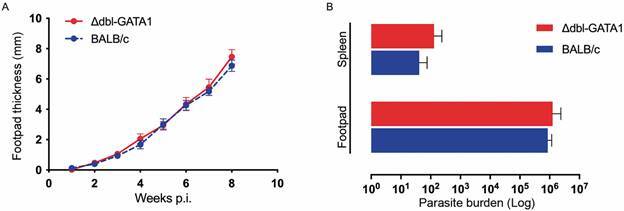
infection of Δdbl-GATA1 with *Leishmania amazonensis*. Footpad thickness of Δdbl-GATA1 and BALB/c mice infected with *L. amazonensis* by week post-infection (A). Parasite load in the spleen and footpad of Δdbl-GATA1 mice and BALB/c mice, infected with *L. amazonensis*. Bars represent mean and standard deviation of log transformed data (B). Data representative of two independent experiments (n = 5 per group).

**Fig. 2:**
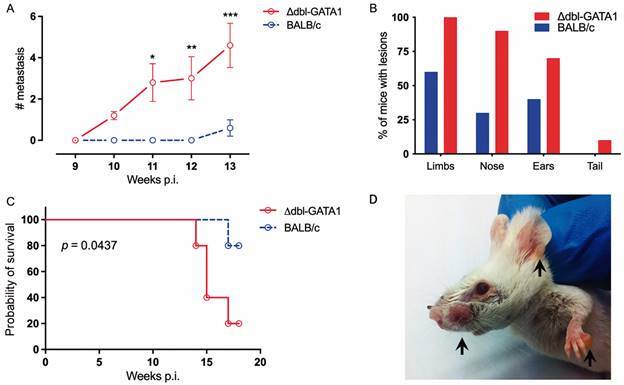
early development of metastatic lesions in Δdbl-GATA1 mice. Number of ectopic lesions found in both strains nine weeks post-infection represented by the total number of cutaneous lesions found in inspection per animal. Bars represent the mean number of metastatic lesions and standard deviation. Statistical differences were measured by Mann-Whitney test (A). Overall percentage of mice with ectopic lesions for anatomic region in the week 13 of infection (B). Kaplan-Meier survival curve of mice infected with *Leishmania amazonensis* (C). Representative figure of lesions in Δdbl-GATA1 mice highlighted by the black arrows. Infection in the lesions was confirmed by histology (D). Data representative of two independent experiments (n = 5 per group).

No differences were observed in lesion development in the footpad between Δdbl-GATA1 mice and BALB/c up to eight weeks after infection with *L. amazonensis* (Fig. 1A). Also, no differences were found in parasite load among mice lineages at the same time of infection (Fig. 1B). After eight weeks, lesions’ size did not increase in size, reaching a plateau. For this reason, no measurement was done after eight weeks. By histology, no significant difference was observed comparing both strains in the liver, spleen, lungs, kidneys, lymph nodes, and footpad. Footpads and lymph nodes displayed a complete structure disruption due to the infiltration of extremely vacuolised macrophages infected with amastigotes [Supplementary data (Fig. 1)]. Surprisingly, we found that metastatic lesions, here defined as new ulcers or nodules in the limbs, head, and tail, began to appear earlier in eosinophil-deficient mice (Fig. 2A). From nine weeks post-infection (wpi), we found a massive growth of metastatic lesions in different body parts, starting in the limbs and then reaching the head and the tail. By 13 wpi, all Δdbl-GATA1 mice had at least one metastatic lesion, and at this time, most of the lesions were observed in limbs, nose, ear, and later, mice developed several lesions all over the tail (Fig. 2B). After 17 wpi, 80% of the Δdbl-GATA1 mice died due to infection, against 20% of deaths in infected BALB/c (Fig. 2C). By 18 wpi, all reminiscent mice were euthanised.

**Fig. 3:**
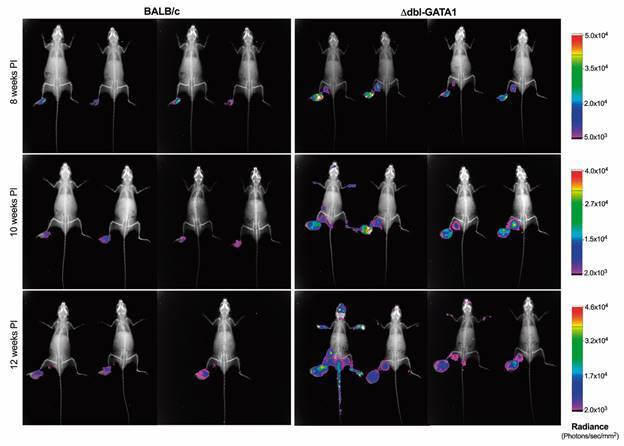
worst outcome of infection in Δdbl-GATA1 mice. Aspect of the metastatic lesions in Δdbl-GATA1 and BALB/c mice at 8-, 10- and 12-weeks post infection with *Leishmania amazonensis-*LUC. X-ray images were overlayed with luciferase signal represented by photons/second/mm^2^. Data representative of one experiment (n = 4 per group).

To increase the sensibility of tracking new metastatic lesions, mice infected with the *L. ama*-LUC strain were monitored in weeks 8, 10 and 12 by whole-body imaging. By week eight, a stronger luciferase signal is observed in the lymph nodes of Δdbl-GATA1 mice compared with very low or absence of signal in BALB/c (Fig. 3). By 10 wpi, several skin lesions might be observed in Δdbl-GATA1 mainly in the extremities and collocated along with the superficial lymph nodes. By 12 wpi, Δdbl-GATA1 is severely affected by the infection with several nodular and ulcerated lesions in the skin (Fig. 2D) replete with parasites as revealed by the luciferase signal, while BALB/c only begun to show signal in the lymph nodes draining the footpad (Fig. 3).

**Fig. 4:**
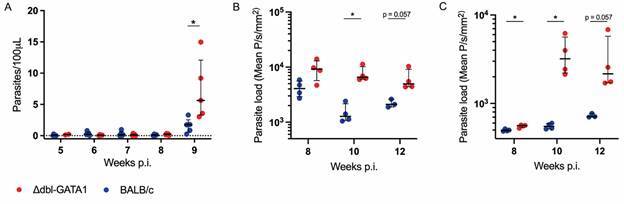
worst outcome of infection in Δdbl-GATA1 mice. Parasite load measured by quantitative polymerase chain reaction (qPCR) in blood samples from Δdbl-GATA1 and BALB/c mice (A). Relative parasite loads in the footpad (B) and draining lymph node (C), measured by signal intensity of luciferase activity in the regions of interest. Bars represent median with interquartile range. Statistical differences were measured by Mann-Whitney test. *p < 0.05. Data representative of one experiment (n = 4 per group).

The pattern of luciferase signal in these experiments suggests that the dissemination of *L. amazonensis* occurs through the lymphatic system. To evaluate whether the dissemination of *L. amazonensis* could also happen through blood, samples were collected from weeks five to nine, and parasite load was measured. No differences in the parasite load in blood were found before nine weeks, comparing both mice strains. At 9 wpi, parasitaemia in Δdbl-GATA1 mice was higher than in BALB/c (Fig. 4A), suggesting that dissemination of amastigotes occurs late in the disease and might depend on the overall parasite burden. After 10 wpi, the parasite load was higher in both footpads (Fig. 4B) and draining lymph nodes (Fig. 4C) of Δdbl-GATA1 mice than BALB/c, as measured by the luciferase signal intensity.

## DISCUSSION

Although eosinophils are found in the initial inflammatory milieu after *Leishmania* infection, their ability to interfere with immune responses remains obscure. Eosinophils are not frequently reported in histological sections of human-infected skin, although they are found in lymph nodes during murine and human tegumentary leishmaniasis.^18^ ^,^ ^19^ In BALB/c mice infected with *L. amazonensis*, a discrete inflammatory infiltrate in the skin is observed in the first weeks after infection, comprised of eosinophils, lymphocytes, and rare parasitised macrophages. During later infection, the dermis showed necrotic tissue, high numbers of mononuclear cells, and vacuolated macrophages filled with amastigotes and hyperplasic germinal centres in the draining lymph nodes. After three months, multifocal inflammatory infiltrates can be observed in the spleen, kidney, and liver.^20^

Although BALB/c mice are highly susceptible to *L. amazonensis* infection, partial immunity has been reported in immunised mice.^21^ In this context, parasite control was associated with close contact between infected macrophages and lymphocytes or eosinophils. Eosinophils were considered exclusively effector cells for a long time, and new data indicates that these cells can affect adaptive immune responses by secreting several cytokines.^11^ ^,^ ^22^ ^,^ ^23^ In addition, the role of promastigotes in releasing eosinophil granules and their leishmanicidal activities are unknown.

Eosinophils have long been associated with tissue damage in allergic disorders. Recently, they gained focus in cancer studies that show their ability to modulate the immune response in the tumoural microenvironment. Several studies suggest an anti-tumorigenic role by exerting cytotoxic activity and suppressing metastasis in melanomas and colorectal cancer.^24^ ^,^ ^25^ Consistent with that observation, we found that in Δdbl-GATA1 mice, infection with *L. amazonensis* was associated with a worse outcome, higher parasite burden, and dissemination of amastigotes.

Metastatic lesions were not an exclusive feature of Δdbl-GATA1 mice since similar nodules were also observed in BALB/c mice, but in this case, lesions began to appear late in physiopathology progress, as the first mice died on week sixteen post-infection. Interestingly, the first metastatic lesions in the skin appeared in the uninfected hindlimb or the forelimbs. However, using an *L. ama*-LUC strain revealed the dissemination pattern that follows the lymphatic vessels weeks before the first since lymph nodes that drain the footpad were the first dissemination site. The appearance of metastatic lesions on the forelimbs and head as soon as nine weeks post-infection suggests anticipated dissemination of amastigotes via bloodstream, as confirmed by qPCR.

Previous assays done by our group showed the same phenotype of early metastatic development in Δdbl-GATA1 when infected with the *L. major* Friedlin strain [Supplementary data (Fig. 2)]. It has been shown that eosinophils are a critical source of IL-4 during the early events after infection, inducing the survival of tissue-resident macrophages that act as a replicative host for *L. major*.^26^ This crosstalk suggests that eosinophils might worsen the infection by promoting a type-2 polarised environment. However, others have found that increased numbers of eosinophils due to increased IL-5 production correlate with lower development of lesions due to *L. amazonensis* infection.^27^ In mice and dogs, but apparently not in humans, eosinophils are substantially recruited by sand flies’ bites and saliva.^10^ ^,^ ^12^ ^,^ ^28^ These findings suggest that eosinophils might have a role in the initial steps of the infection with *Leishmania* spp. Recently, eosinophils have been showed to act as by-standing cell promoting parasite killing via paracrine secretion of PGD_2_, inducing macrophages to kill amastigotes of *L. amazonensis in vitro*.^29^ Thus, we hypothesise that eosinophils might exert a direct leishmanicidal activity at the site of infection since they are massively recruited by salivary components of sand flies and/or by modulating the immune system towards a resistance phenotype.

Although our model suggests that the lack of eosinophils might represent a defect in parasite control, some limitations must be considered. First, it has been extensively reported that Δdbl-GATA1 mice display functional deficits during inflammation since GATA1 regulates numbers of hematopoietic stem progenitor cells in the bone marrow.^30^ In addition, since GATA1 is associated with hematopoiesis,^31^ they display both numerical and functional aberrancy in basophils,^32^ defects in platelet biology,^33^ and mast cell maturation.^34^ Thus, the phenotype observed cannot be attributed solely to the lack of eosinophils. Our model showed the dissemination of *L. amazonensis* and could help us understand the dissemination of other species that cause mucocutaneous or visceral leishmaniasis, such as *L. braziliensis* and *L. guyanensis*, and *L. infantum*, respectively. Further studies might address the role of eosinophils in different contexts of infection and in resistant strains of mice lacking eosinophils.
