# Supplementary material for: The absence of eosinophils is associated with early metastatic lesions in Leishmania amazonensis-infected mice
Source: Mem Inst Oswaldo Cruz. 2024 Jan 8;119:e220242. doi: 10.1590/0074-02760220242 (PMC10777375; doi:10.1590/0074-02760220242)
Supplement: Supplementary file 1 [file 1678-8060-mioc-119-e220242-s.pdf]

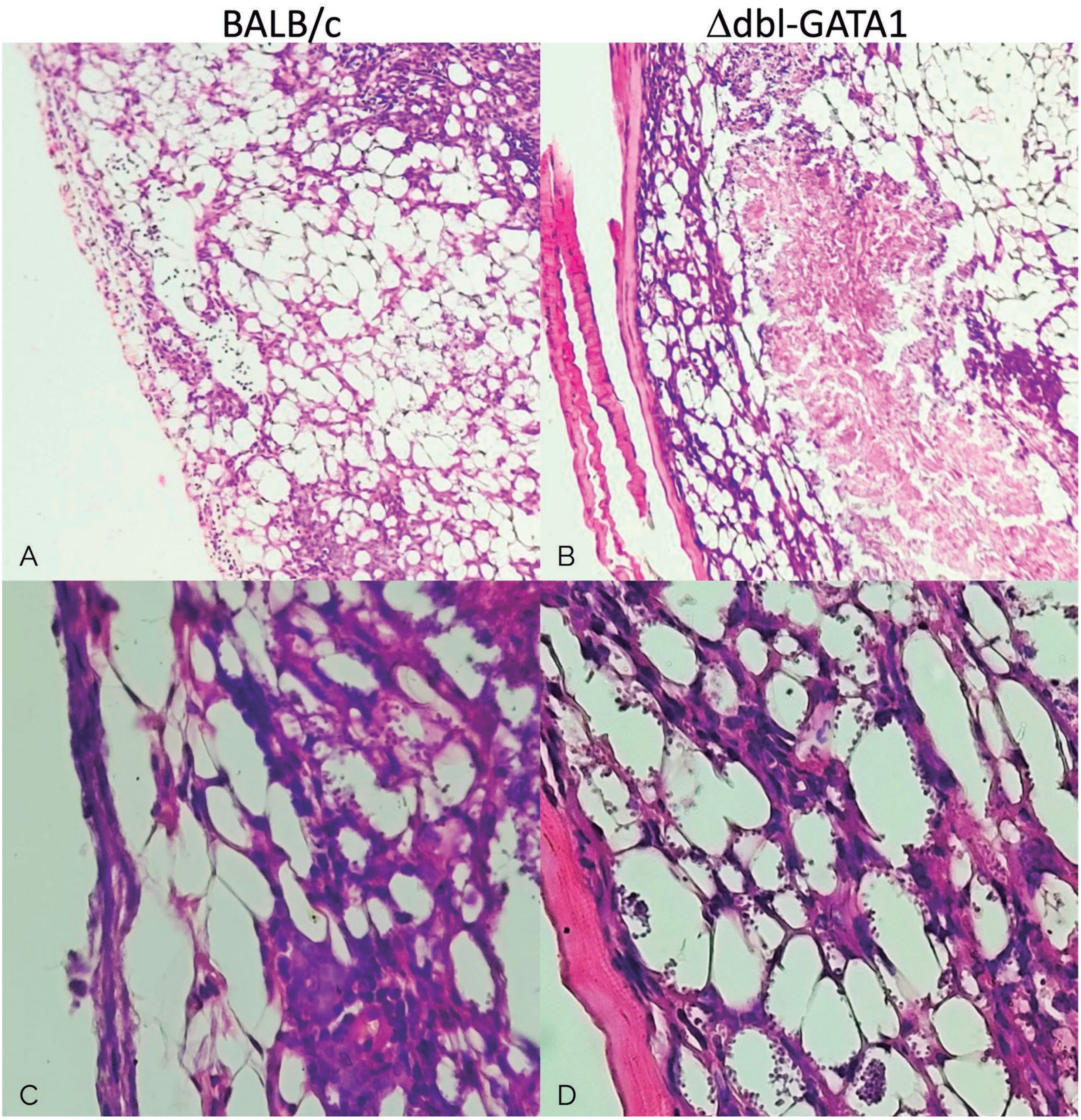

Fig. 1: representative histology of lymph nodes from BALB/c mice and Δdbl-GATA1 infected with *Leishmania amazonensis*. Representative images in light microscopy of histologic slides of lymph nodes at eight weeks post-infection with *L. amazonensis*. BALB/C (A and C) and Δdbl-GATA1 (B and D) mice at 10x (A and B) and 40x (C and D) magnification.

CC BY

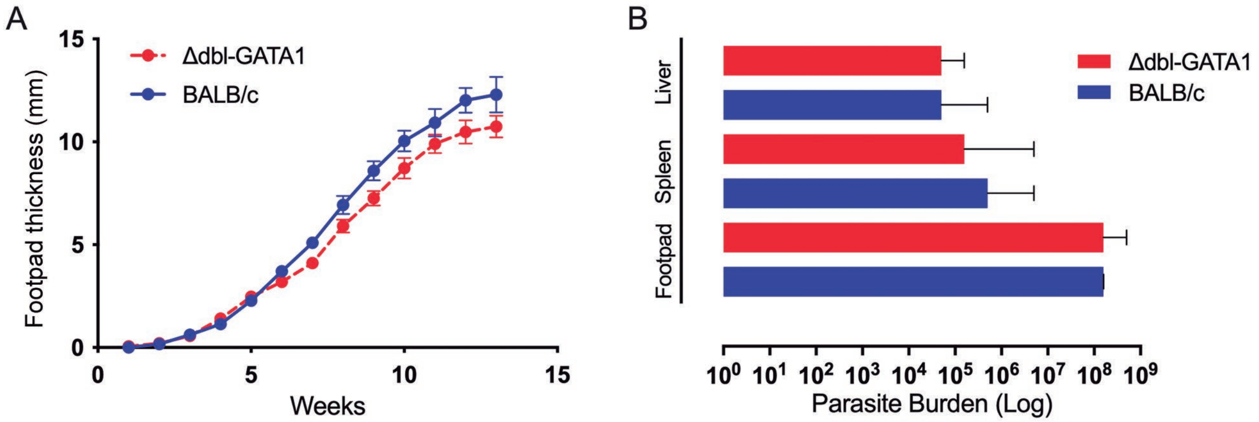

Fig. 2: infection of Δdbl-GATA1 with *Leishmania major* (Friedlin). Footpad thickness of Δdbl-GATA1 and BALB/c mice infected with *L. major* by week post infection (A). Parasite load in the spleen, liver, and footpad of Δdbl-GATA1 mice and BALB/c mice, infected with *L. major* 13 weeks post-infection. Bars represent mean and standard deviation of log transformed data (B). Data representative of two independent experiments (n = 5 per group).
